# Predictors of Short- and Long-Term Mortality in Males and Females with Hip Fracture - A Prospective Observational Cohort Study

**DOI:** 10.1371/journal.pone.0078169

**Published:** 2013-10-29

**Authors:** Andreas P. Diamantopoulos, Mari Hoff, Marc Hochberg, Glenn Haugeberg

**Affiliations:** 1 Department of Rheumatology, Hospital of Southern Norway Trust, Kristiansand, Norway; 2 Department of Neurosciences, Faculty of Medicine, Norwegian University of Science and Technology, Trondheim, Norway; 3 Department of Public Health and General Practice, Faculty of Medicine, Norwegian University of Science and Technology, Trondheim, Norway; 4 Departments of Medicine, Epidemiology and Preventive Medicine, University of Maryland School of Medicine, Baltimore, Maryland, United States of America; 5 Faculty of Health and Sport Sciences, University of Agder, Kristiansand, Norway; 6 Department of Rheumatology, St Olavs Hospital, Trondheim, Norway; Children’s National Medical Center, Washington, United States of America

## Abstract

**Background:**

Hip fracture is associated with increased mortality. Our aim was to study potential risk factors, including osteoporosis, associated with short- and long-term mortality in a prospectively recruited cohort of fragility hip fracture patients.

**Methodology/Principal Findings:**

Fragility hip fracture patients aged >50 years admitted to a county hospital in Southern Norway in 2004 and 2005 were consecutively identified and invited for assessment. Patients with high energy or pathological fractures, patients with confusion, serious infections or who were non-residents in the catchment area were excluded. As part of a clinical routine, data were collected using questionnaires. Standardized bone density measurements of lumbar spine and hip were performed. Potential predictors of hip fracture mortality were tested using univariate and multivariate logistic regression analysis. A total of 432 hip fracture patients (129 males and 303 females) were prospectively identified. Among them 296 (85 males and 211 females) patients [mean age 80.7 (SD 9.1)] were assessed at the Osteoporosis center. Variables independently associated with short-term mortality (after 1 year) were in females older age [Odds Ratio (OR) 6.95] and in males older age (OR 5.74) and pulmonary disease (OR 3.20), whereas no associations were observed with mortality for 3 months after the fragility hip fracture. Variables independently associated with 5 years mortality in males was osteoporosis (OR 3.91) and older age (OR 6.95), and in females was dementia (OR 4.16) and older age (OR 2.80).

**Conclusion:**

Apart from known predictors as age and comorbidity osteoporosis in our study was identified as a potential independent predictor of long-term hip fracture mortality in males. This is of particular importance as treatment with bisphosphonates after hip fracture has been shown to reduce hip fracture mortality and may be a clinical target to reduce the burden of the disease. Further studies however are needed to confirm the validity of this finding.

## Introduction

Several studies have shown that mortality in hip fracture patients in the middle-aged and elderly population is higher compared to the general population and in males than in females [1]–[3]. This increased mortality in hip fracture patients has been documented in the first year after fracture and remains high in the subsequent years of follow up [3].

Understanding which risk factors for mortality are involved in hip fracture patients is crucial in order to reduce mortality in this group. Pre-fracture conditions have been reported to influence the mortality and morbidity in fragility hip fracture patients [4]. The identified predictors of mortality include older age at fracture, male gender [5]–[8], poor mental condition [7], [9], pre-fracture comorbidities (e.g. cardiovascular and pulmonary diseases) [5], [6], [10], fracture site [9], complications during surgery [9], and limited function [7]. A recent systematic review and meta-analysis concluded that advanced age, male gender, nursing home residence, poor preoperative walking capacity and activities of daily living, poor mental status, dementia or cognitive impairment, diabetes, cancer, cardiac disease and presence of multiple comorbidities, are strongly associated with short-term increased mortality in patients suffering a fragility hip fracture [11]. However, these studies only explored risk factors for short and not for long-term hip fracture mortality.

Reduced bone mineral density (BMD) has been shown to be strongly associated with increased risk for hip fracture [12] and interestingly treatment with bisphosphonates in hip fracture patients has been shown to reduce subsequent hip fracture mortality [13]. However, to our knowledge, no studies have examined the influence of osteoporosis as a risk factor for hip fracture mortality neither in males nor in females both in the short- and long-term aspect. Thus, identifying osteoporosis as a potential risk factor for mortality after hip fracture could be of great value by detecting patients at high risk of mortality.

In this study, our primary aim was to explore for short- and long- term risk factors of mortality in hip fracture patients in a prospectively recruited cohort of patients followed for up to 5 years. In addition, we also explored the potential role of osteoporosis as a risk factor for mortality after hip fracture.

## Methods

### Ethics

The study was approved by the Regional Committee for Medical Research Ethics Southern-Eastern Norway. Informed written consent was obtained by all the participants in this study.

### Identification of the Fragility Hip Fracture Patients

Patients with a fragility hip fracture aged 50 years or older admitted to the Hospital of Southern Norway in Kristiansand in the two year period 2004 through 2005 were prospectively identified by trained osteoporosis nurses at our osteoporosis center. The osteoporosis center is organized according to the fracture liaison service model [14]. The identified patients’ medical records and X-ray records were examined, and the diagnosis of hip fracture was confirmed before being invited for osteoporosis assessment as part of the clinical program for these patients. Patients with high energy fractures, e.g. due to motor vehicle accident, or with pathological fractures, e.g. caused by tumor, were excluded. We also excluded patients with confusion, serious infection and who were non-residents in the catchment area.

Patient recruitment, data collection and assessments as well as dual-energy x-ray absorptiometry (DXA) measurements were performed by four trained and experienced in osteoporosis nurses.

### Mortality

The follow-up time for a patient was from the month that the fracture occurred to death. For patients alive, the censoring dates were on January 1, 2009 (recruited in 2004) and 2010 (recruited in 2005). Mortality was determined by matching the death data to those of the Norwegian census. No data on death causes was collected.

### Demographic and Clinical Variables

Data collection included clinical assessments, standardized questionnaires and BMD measurements (age, gender, body mass index (BMI), osteoporosis, type of fracture, type of fracture treatment, involvement of snow/ice, outdoors fracture, patient discharged to home, impaired vision, cardiovascular disease, pulmonary disease, gastrointestinal disease, kidney disease, urogenital disease, cancer, inflammatory joint disease, diabetes mellitus, other endocrinopathies, dementia, stroke, other neurological disease, depression, other mental disease, alcoholism, use of sedatives, osteoporosis treatment prior to fracture).

An overview of the variables studied is presented in Table 1.

**Table 1:** Baseline characteristics of 296 patients assessed at the osteoporosis center suffering a fragility hip fracture in 2004 and 2005.

Characteristics	All patients (n = )	Males (n = )	Females (n = )	P values
**Age (years)**	80.6 (9.1)	80 (8.9)	81 (9.2)	0.47
**Gender**	296	85 (28.7)	211 (71.3)	
**>80 years**	173	46 (54.1)	127 (60.0)	0.33
**Body mass index kg/m^2^**	22.9 (3.7)	23 (3.6)	22.7 (3.8)	0.21
***Osteoporosis***	***218 (73.6)***	***53 (62.4)***	***165 (78.9)***	***<0.01***
**Type of hip fracture**				
Neck	200 (67.6)	62 (72.9)	138 (65.4)	0.134
Per-trochanteric	88 (29.7)	23 (27.1)	65 (30.8)	
Sub-trochanteric	8 (2.7)	0	8 (3.8)	
**Type of treatment**				
Not operated	10 (3.4)	3 (3.5)	7 (3.3)	0.20
CHP	163 (55.1)	54 (63.5)	109 (51.6)	
DHS	107 (36.1)	26 (31.0)	81 (38.3)	
Total or hemi-prosthetic joint	16 (5.1)	2 (2.4)	14 (6.7)	
**Cardiovascular disease**	145 (49.0)	40 (47.1)	105 (50.0)	0.64
**Pulmonary disease**	46 (15.5)	17 (20.0)	29 (13.8)	0.18
**Gastrointestinal disease**	37 (12.5)	14 (16.5)	23 (11.0)	0.19
***Kidney disease***	***7 (2.4)***	***6 (7.1)***	***1 (0.5)***	***<0.01***
**Inflammatory joint disease**	27 (9.1)	6 (7.1)	21 (10.0)	0.43
**Diabetes mellitus**	18 (6.1)	7 (8.2)	11 (5.2)	0.32
**Depression**	14 (4.7)	3 (3.5)	11 (5.2)	0.53
**Endocrinopathies (other)**	35 (11.8)	10 (11.8)	25 (11.9)	0.97
**Neurological disease (other)**	72 (24.3)	22 (25.9)	50 (23.8)	0.70
***Urogenital disease***	***26 (8.8)***	***17 (20.0)***	***9 (4.3)***	***<0.01***
**Dementia**	30 (10.1)	8 (9.4)	22 (10.5)	0.78
**Stroke**	29 (9.8)	9 (10.6)	20 (9.5)	0.77
**Mental disease (other)**	14 (4.7)	3 (3.5)	11 (5.2)	0.53
**Cancer**	39 (13.2)	15 (17.6)	24 (11.4)	0.15
**Alcoholism**	3 (1.0)	2 (2.4)	1 (0.5)	0.14
***Sedatives***	***50 (16.9)***	***9 (10.6)***	***41 (19.4)***	***0.06***
***Snow/ice involved***	***45 (15.2)***	***24 (28.2)***	***21 (10.0)***	***0.01***
***Outdoors fracture***	***89 (30.1)***	***34 (40.0)***	***55 (26.1)***	***<0.01***
**Discharged at home**	89 (30.1)	30 (35.7)	59 (28.0)	0.19
**Impaired vision**	9 (3.0)	3 (3.5)	6 (2.9)	0.76
**Osteoporosis treatment**	18 (6.1)	4 (5.6)	14 (8)	0.50

Continuous variables are presented as mean with standard deviation (SD) and categorical variables as numbers and percentage. For group comparison independent sample t-tests were applied for continuous variables and chi-square tests for categorical variables.

SD: Standard deviation, CHP: Cannulated hip screw DHS: Dynamic hip screw.

### Bone Density Measurements

Standardized BMD measurements at lumbar spine L2–4, femoral neck and total hip on the non-fractured hip were performed by four trained nurses by using DXA equipment, Lunar Prodigy (General Electric) at baseline. The BMD measurements were expressed as T-scores (SD) with calculations based on the reference values in the DXA machine provided by the manufacturer. Osteoporosis was defined as T -score ≤−2.5, osteopenia as T-score>−2.5 and <−1.0, and normal BMD a s T-score ≥−1.0, according to the WHO definition for osteoporosis at spine and/or at hip [15].

### Statistical Methods

The Kaplan-Meier method was used to estimate the probability of survival 5 years after the fragility hip fracture. Intergroup comparisons were performed using the log-rank test. For group comparisons we applied the chi square test for categorical variables and the Students t-test for continuous variables. Exploring for predictors of mortality at 3 months, 1 and 5 years after the hip fracture event, we first applied a univariate regression model. Variables tested in the univariate model with a p value <0.1 were then tested by adjusting for age and thereafter tested in multivariate logistic regression using an entry procedure. A logistic regression analysis model was applied to search for significant predictors of mortality in the fragility hip fracture patients with death and age as the dependent variables, and statistically significant variables at the 0.1 level in the bivariate analysis as the explanatory variables. The analysis was performed separately for males and females. All the analyses were performed using the SPSS version 17.0 (SPSS, Chicago, IL, USA). Statistical significance was defined as p<0.05.

### Funding

This work has been supported and funded by the Competence Development Fund of Southern Norway and the Hospital of Southern Norway Trust.

## Results

### Baseline Characteristics

We prospectively identified 432 individuals who suffered a fragility hip fracture in the catchment area of the hospital in the two years 2004 and 2005. One hundred and thirty six patients [mean age 84 years (SD 7.9], 44 males [mean age 83.7 (SD 9.1)] and 92 females [mean age 84.3 (SD 7.3)] were not assessed at the osteoporosis center of Kristiansand Hospital due to severe confusion or serious infection. The 296 patients [mean age 80.7 (SD 9.1)] who were assessed at the osteoporosis center included in this study, 86 males [mean age 80.0 years (SD 8.9)] and 211 females [mean age 80.9 years (SD 9.2)] were interviewed, examined by the trained nurses and measured by DXA. Patients not assessed at the osteoporosis center were in mean 3.5 years older (p<0.0005) [males 3.7 years (p = 0.014) and females 3.1 years (p = 0.006)]. There were no significant gender differences between patients assessed and not assessed at the osteoporosis center (29% vs. 32% for males and 71% vs. 68% for females, p = 0.443).The baseline characteristics of the assessed patients are presented in Table 1.

### Mortality of the Fragility Hip Fracture Patients

**Figure 1:**
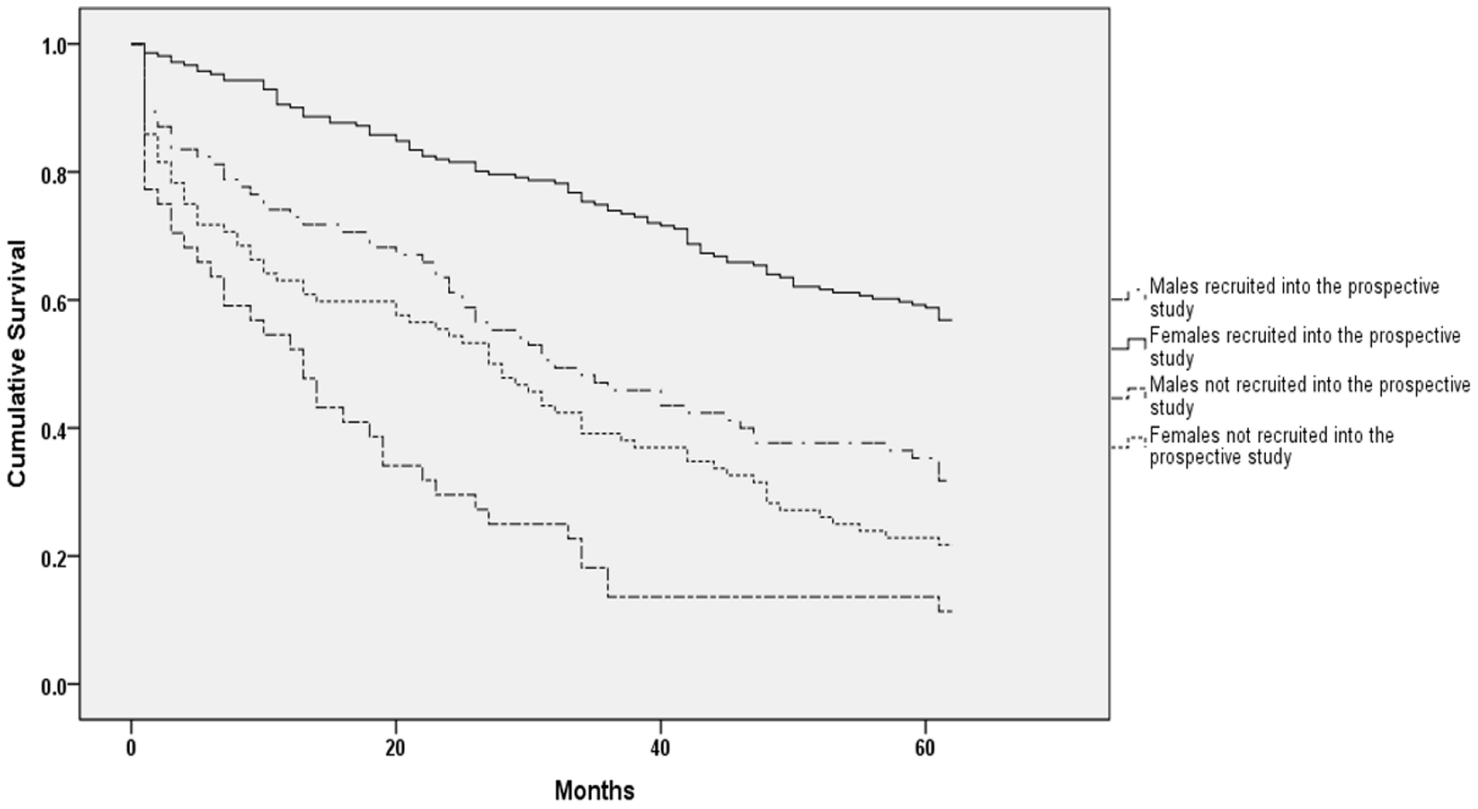
Kaplan-Meier survival curves of male and female hip fracture patients prospectively recruited into the prospective osteoporosis cohort compared to those excluded from the study.

Three months after the hip fracture event 12 males (14.1%) and 5 females (2.1%) were dead (p<0.01) and after one year 22 males (25.9%) and 21 females (10.0%) were dead (p<0.01). Five years after the fragility hip fracture 60 males (70.6%) and 102 females (48.3%) in the cohort group had died (p<0.01). For the patients not assessed at the osteoporosis center the corresponding death figures were for 3 months, 1 year and 5 years follow up 13 (29%), 26 (59%) and 39 (87%) for males and 18 (24%), 37 (40%) and 75 (81%) for females. The log-rank analysis showed a statistically significant difference between the two groups in both males and females (p<0.001) (Fig. 1).

### Univariate Analysis and Analysis Adjusted for Age

In the univariate analysis, for the short- term mortality (3 months), osteoporosis treatment in females and dementia, gastrointestinal diseases, no involvement of snow or ice, indoor activity, impaired vision, stroke and age >80 years in males were significantly associated with death. For short-term mortality (1 year) the associations included indoor activity, osteoporosis treatment, and age >80 years in females and discharge to home, indoor activity, stroke, neurologic disease, pulmonary disease and no involvement of snow or ice in males. Variables significantly associated with long-term mortality (5 years) included no involvement of snow or ice, indoor activity, osteoporosis, dementia, visual impairment, use of sedatives, discharge to home and age >80 years for females and no involvement of snow or ice, indoor activity, osteoporosis, dementia, cancer stroke and age in males. No associations were observed with regard to cardiovascular diseases, diabetes mellitus, other endocrinopathies, depression, other mental diseases, alcoholism, type of fracture, type of fracture treatment, BMI, weight and BMD of spine (L2–L4), and right or left hip. In the age-adjusted univariate analysis of 5 years associations, variables significantly associated were no involvement of snow or ice and osteoporosis in males, and in females no involvement of snow or ice, dementia, use of sedatives and indoor activity, as shown in table 2 and 3.

**Table 2:** Univariate, age-adjusted, and multivariate logistic regression analysis showing associations between the different factors and the dependent variable of the study (mortality) in males 5 years after the hip fracture.

	Univariate model		Age adjusted		Multivariate model	
	P values	OR (95% C.I.)	P values	OR (95% C.I.)	P values	OR (95% C.I.)
**Age (ref group<80)**	**<0.0005**	**8.63 (2.81–26.47)**	**–**	**–**	**0.004**	**6.95 (1.88–25.58)**
Stroke (ref. group no)	0.041	1.17 (1.05–1.30)	0.99	N/A	0.99	N/A
Cancer (ref. group no)	0.033	7.30 (0.90–58.93)	0.08	6.9 (0.8–61.3)	0.53	2.08 (0.20–20.73)
Dementia (ref. group no)	0.055	1.15 (1.04–1.27)	0.99	N/A	0.99	N/A
Snow/ice involved (ref. group no)	<0.0005	0.44 (0.23–0.84)	0.006	0.22 (0.06–0.63)	0.053	0.13 (0.01–1.01)
Outdoor fracture (ref. group no)	0.004	0.24 (0.09–0.64)	0.06	0.35 (0.12–1.05)	0.36	2.44 (0.35–16.97)
**Osteoporosis (ref. group no)**	**0.001**	**4.88 (1.80–13.25)**	**0.01**	**4.2 (1.40–12.63)**	**0.03**	**3.91 (1.08–14.08)**

OR: odds ratio, C.I.: confidence interval, ref.: reference.

**Table 3:** Univariate, age-adjusted, and multivariate logistic regression analysis showing associations between the different factors and the dependent variable of the study (mortality) in females 5 years after the hip fracture.

	Univariate model		Age adjusted		Multivariate model	
	P values	OR (95% C.I.)	P values	OR (95% C.I.)	P values	OR (95% C.I.)
**Age (ref group<80 years)**	**<0.0005**	**3.63 (2.01–6.54)**	**–**	**–**	**0.003**	**2.80 (1.43–5.48)**
Discharged home (ref. group no)	0.021	0.48 (0.26–0.90)	0.13	0.60 (0.31–1.16)	0.47	0.77 (0.37–1.57)
**Dementia (ref. group no)**	**0.001**	**5.57 (1.81–17.09)**	**0.007**	**4.90 (1.56–15.65)**	**0.01**	**4.16 (1.29–13.45)**
Impaired vision (ref. group no)	0.084	5.51 (0.63–48.04)	0.18	4.50 (0.49–41.33)	0.17	4.70 (0.50–43.68)
Snow/ice involved (ref. group no)	0.001	0.79 (0.68–0.92)	0.01	0.20 (0.05–0.75)	0.44	0.55 (0.12–2.48)
Outdoor fracture (ref. group no)	<0.0005	0.29 (0.15–0.58)	<0.0005	0.28 (0.14–0.57)	0.09	0.48 (0.21–1.13)
Osteoporosis *(ref. group no)*	0.005	2.72 (1.33–5.57)	0.09	1.91 (0.89–4.09)	0.24	1.63 (0.71–3.74)
Use of sedatives (ref. group no)	0.071	1.88 (0.94–3.78)	0.05	2.08 (0.99–4.35)	0.07	2.02 (0.92–4.44)

OR: odds ratio, C.I.: confidence interval, ref.: reference.

### Multivariate Logistic Regression Analysis

In the logistic regression analysis model, no associations were observed neither for females or males for the 3 months after the fragility hip fracture (data not shown). Variables associated with short-term mortality (1 year) were age >80 years in females (OR 6.95) and age >80 years (OR 5.74) and pulmonary disease (OR 3.20) in males. Variables associated with 5 years mortality were age >80 years, dementia in females (table 2) and age >80 years and osteoporosis in males (Table 3).

## Discussion

In our study, aiming to identify predictors of mortality in hip fracture patients, older age and comorbidities was found to be associated with increased risk of death both after 1 and after 5 years of follow up. However, none of the examined potential risk factors was found to be independently associated with death after 3 months when tested in multivariate analysis, not even age. We found osteoporosis to be associated with 5 years mortality in univariate analysis for both men and women. When adjusting for age, osteoporosis remained statistically significant for males and was borderline significant associated with increased risk of death in females. In men but not in women, osteoporosis even remained independently associated with increased 5 years mortality.

To our knowledge, this is the first study to examine the direct contribution of osteoporosis as a risk factor for mortality in hip fracture patients. This is an interesting finding as treatment with bisphosphonates after hip fracture has been shown to reduce mortality [13] and may be a clinical target to reduce the burden of the disease. Males have higher mortality rates and are in greater risk of institutionalization than females [16]. Hence, the observed 3-fold increase in long-term mortality of the fragility hip fracture, could explain the higher mortality rates and maybe predict a greater efficacy of anti-osteoporosis treatment [17] in this group of patients.

Older age is a strong predictor of mortality in both genders [5]–[8], [11]. It seems that the frail older population is most sensitive to the complications related to the fracture event [18]. As the aging population is steadily growing in Western societies, intense efforts are warranted in order to reduce the number of hip fractures in this group of patients.

In our study, we also found other risk factors associated with increased mortality after hip fracture. Dementia in females was found to be associated with 5 years mortality, increasing the death risk by approximately 4 times (Table 3). Nevertheless, dementia was not associated with increased mortality neither 3 months nor 1 year after the event. This is in agreement with previous published studies, which have shown no association between reduced mental status and the short-term mortality of hip fracture [8], [19]. However, this is in contrast with other studies, showing a clear contribution of the reduced mental status in the mortality rates in females [7], [11]. One explanation could be that we excluded all patients with a severe deterioration of their mental status after hip fracture. Delirium during the perioperative period seems to be associated with severe worsening of cognitive functions and is a strong predictor of short-term mortality in fragility hip fracture patients [8].

With regard to other pre-fracture comorbidities, we found pulmonary disease to be associated with increased mortality in males and this was only one year after fracture. This has also been described elsewhere [10], [20]. While in some studies a clear association with comorbidities (e.g. cardiovascular) was demonstrated, we were not able to demonstrate any other associations in short or long-term mortality with increased risk of mortality after hip fracture. The above findings explain only partially the increased mortality in males suffering a fragility fracture in short- and long-term aspects. However, prevention and treatment of pulmonary diseases in males should be emphasized in order to reduce the risk of death in short-term. Our data adds evidence that reduced health condition prior to fracture in an individual with a hip fracture increases mortality risk.

In our study, we did not observe any influence of osteoporosis treatment neither in males nor in females both for short- and long-term mortality. This may be explained by the fact that our study including a rather small number of patients and was not designed to investigate the impact of osteoporosis treatment. In other studies, a reduced mortality rate has been reported in patients treated with bisphosphonates [13] [21], [22]. A disappointing finding in our research is that despite osteoporosis being established in up to 79% of females and 62% of males patients only 8% of females and 6% of males received osteoporosis treatment.

We did not observe any influence in hip fracture mortality of the type of fracture or surgical treatment selected neither on males nor on females. This finding is in agreement with previous published studies [10], [23].

### Limitations

Our study has limitations. A significant part of the fragility hip fracture patients (31%) were not assessed at the osteoporosis center. This group was significantly older than and had a higher mortality than patients assessed at the osteoporosis center as shown in Figure 1. Therefore, it is reasonable to believe that the major part of the frailest of patients was not included in our analysis. For that reason, our results could be influenced by selection bias as in our cohort we included the healthiest of the fragility hip fracture patients. The above selection could also explain the lack of association between comorbidities and increased short- and long-term mortality.

*In conclusion,* high age and comorbidities were found to be predictors of mortality in hip fracture patients not only in the first year but also at five years of follow up. Our data indicates that osteoporosis is a risk factor for long-term mortality in hip fracture patients. This finding was more significant in males than in females. Further studies however are needed to confirm the validity of this finding.
